# Supplementary material for: The SIGMA rat brain templates and atlases for multimodal MRI data analysis and visualization
Source: Nat Commun. 2019 Dec 13;10:5699. doi: 10.1038/s41467-019-13575-7 (PMC6911097; doi:10.1038/s41467-019-13575-7)
Supplement: Supplementary file 1 — Supplementary Information [file 41467_2019_13575_MOESM1_ESM.pdf]

The SIGMA rat brain templates and atlases for multimodal MRI data analysis and visualization

D.A. Barrière[1,2,3,†], R. Magalhães[1,2,4,5,†], A. Novais[4,5], P. Marques[4,5], E. Selingue[1,2], F. Geffroy[1,2], F. Marques[4,5], J. Cerqueira[4,5], J.C. Sousa[4,5], F. Boumezbeur[1,2], M. Bottlaender[1,2], T.M. Jay[3,6,7], A. Cachia[3,6,8,9], N. Sousa[4,5,**], S. Mériaux[1,2,*]

[1]NeuroSpin, Institut des Sciences du Vivant Frédéric Joliot, Commissariat à l'Énergie Atomique et aux Énergies Alternatives, 91191 Gif-Sur-Yvette, France.
[2]Université Paris-Saclay, 91191 Gif-Sur-Yvette, France
[3]Physiopathologie des Maladies Psychiatriques, UMR_S 894 Inserm, Centre de Psychiatrie et Neurosciences, 75014 Paris, France.
[4]Life And Health Sciences Research Institute (ICVS), School of Medicine, University of Minho, 4710-057 Braga, Portugal.
[5]ICVS/3B's, PT Government Associate Laboratory, Braga/Guimarães, Portugal.
[6]Université Paris Descartes, Sorbonne Paris Cité, 75006 Paris, France.
[7]Faculté De Médecine Paris Descartes, Service Hospitalo-Universitaire, Centre Hospitalier Sainte Anne, 75014 Paris, France.
[8]Laboratoire de Psychologie du Développement et de l'Éducation de l'Enfant, CNRS UMR 8240, 75014 Paris, France.
[9]Institut Universitaire de France, 75005 Paris, France

[*] Corresponding author: Dr. Sébastien Mériaux
CEA / DRF / JOLIOT / NeuroSpin
CEA Centre de Saclay
Bâtiment 145 - Point Courrier 156
91191 Gif-sur-Yvette CEDEX, France
sebastien.meriaux@cea.fr

[**]Co-corresponding author: Dr Nuno Sousa
School of Medicine
Life and Health Sciences Research Institute (ICVS)
University of Minho
4710-057 Braga, Portugal
njcsousa@med.uminho.pt

[†]These authors contributed equally to this work

Supplementary Information

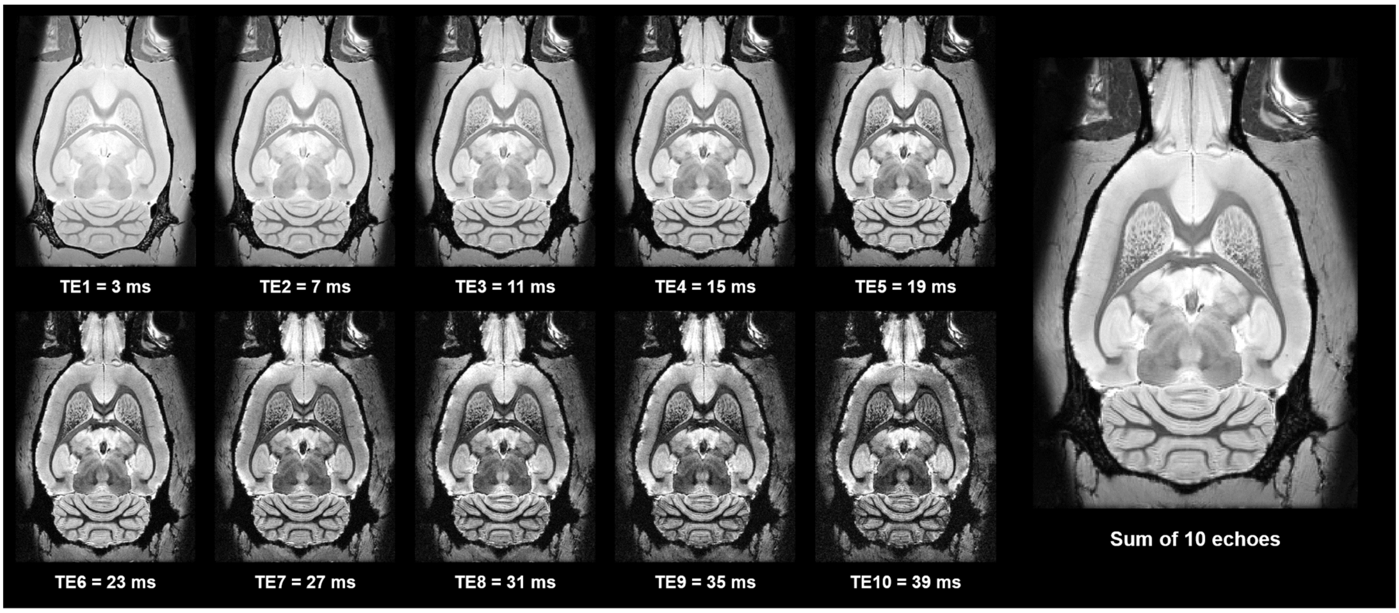

Supplementary Figure 1: Example of an *ex vivo* Multiple Gradient Echo acquisition used to build the SIGMA anatomical template of rat brain (TR = 100 ms, 10 echoes spaced by 4 ms from 3 ms to 39 ms, spatial resolution = 90 x 90 x 180 µm^3, 16 averages, acquisition time = 8 h 32 min).

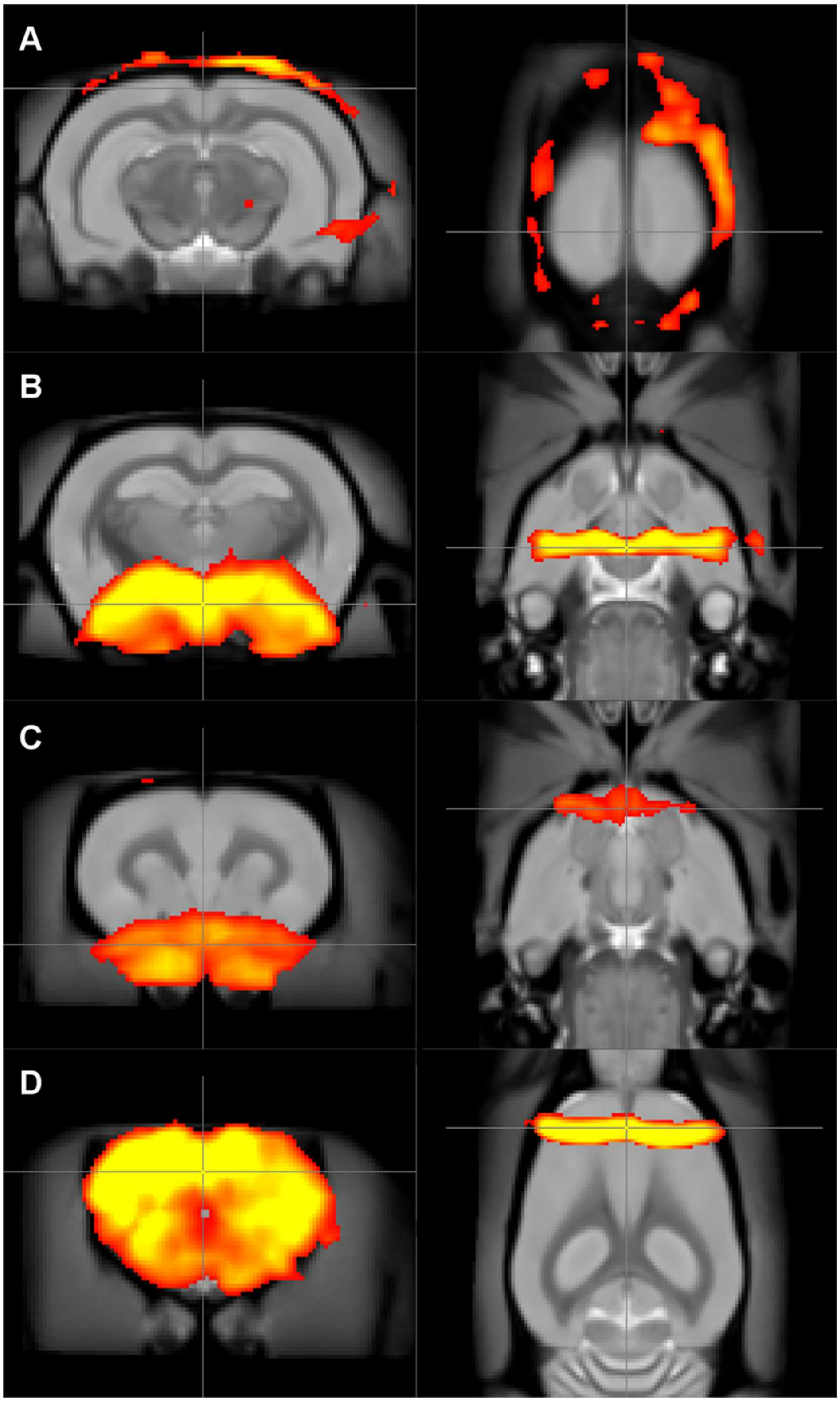

Supplementary Figure 2: Several examples of artefact found through ICA decomposition (left column = coronal slices, right column = axial slices). (**A**) Movement artefact component, (**B** and **C**) ghosting artefact components, (**D**) antenna channels crosstalk artefact.

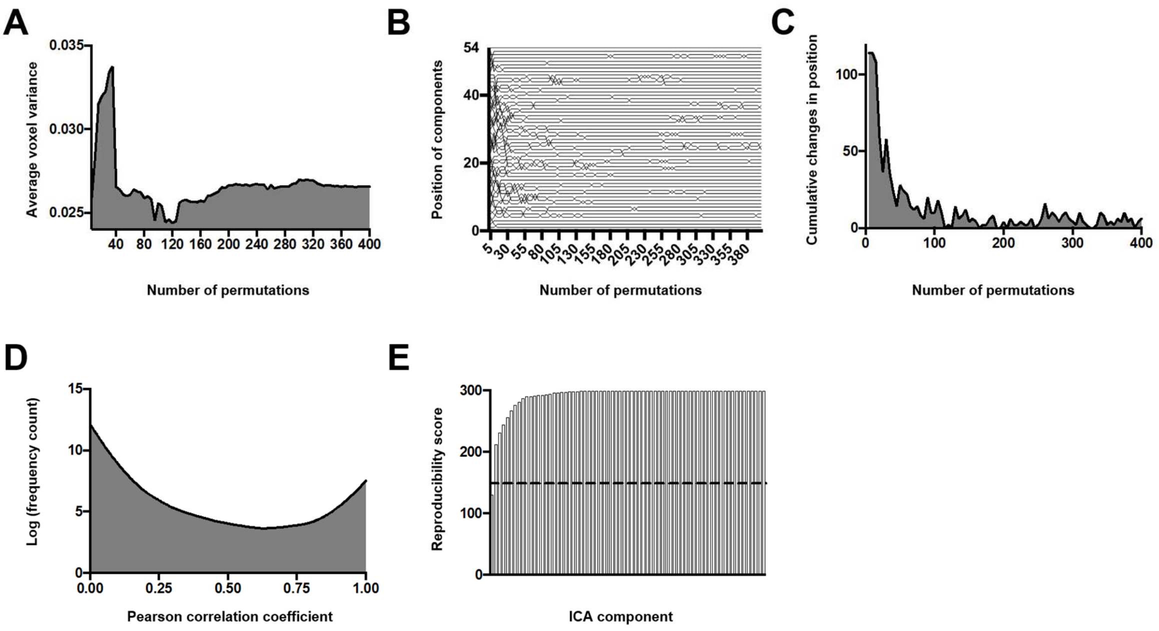

Supplementary Figure 3: Impact of the number of permutations upon the reproducibility of ICA maps. (**A**) Average voxel variance across the grouped maps for each number of permutations used. (**B**) Rank of the reproducibility of each component across each number of permutations. (**C**) Cumulative changes in rank position calculated as the sum of the absolute differences between *i* and *i*–1 permutation. (**D**) Logarithmic distribution of the correlation distribution of all possible pairs for N = 300 permutations. (**E**) Reproducibility of all the components, ordered by reproducibility. In **E**, the dashed line indicates the cut-off threshold, equal to half of the maximum reproducibility found. Source data are provided as a Source Data file.

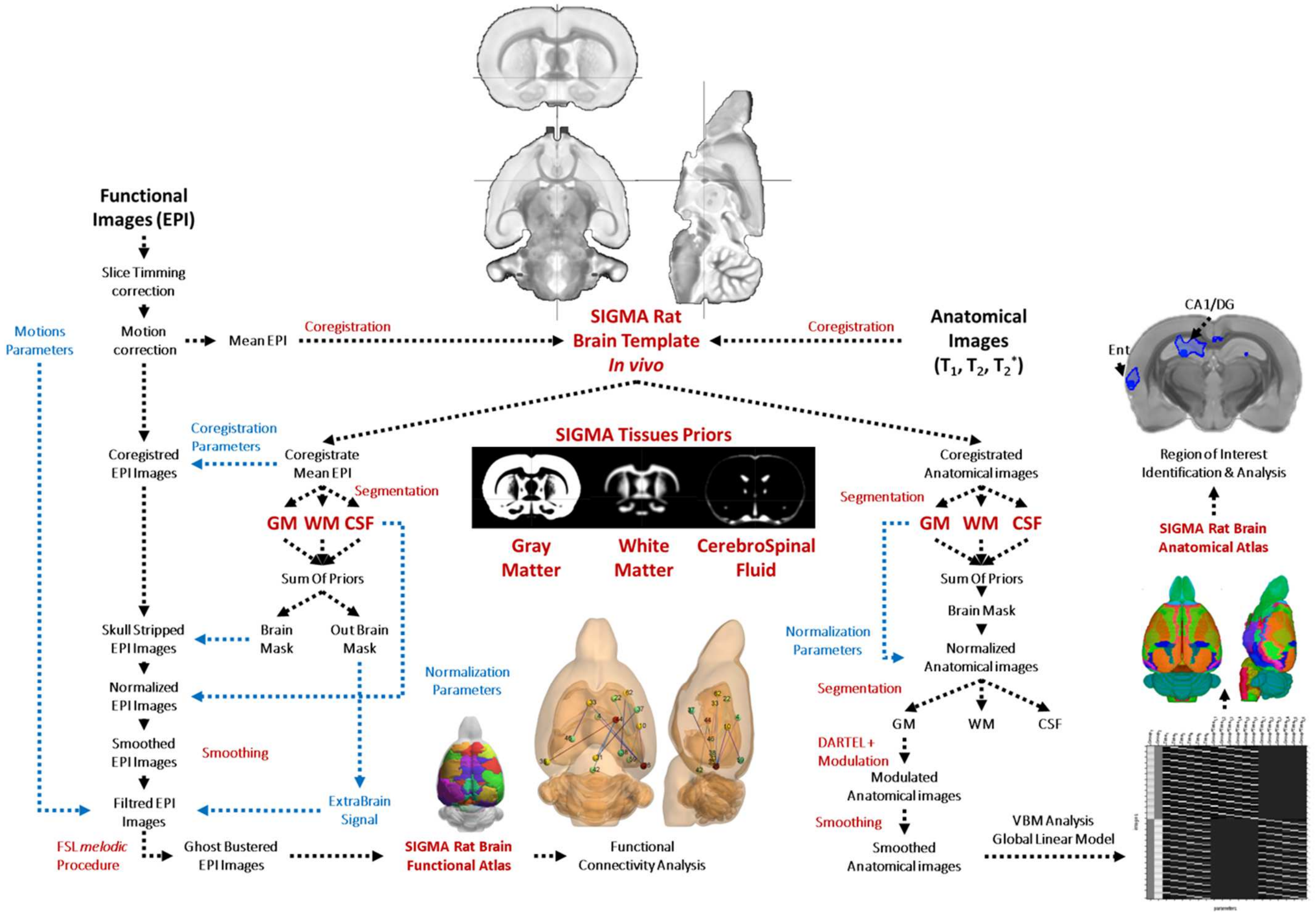

Supplementary Figure 4: The power of the integrated analysis in functional and morphological MRI investigation in rat. The SIGMA resources offer to the preclinical neuroimaging community a complete set of *in vivo* and *ex vivo* templates, tissues priors as well as functional and anatomical atlases of the rat brain. This new set of resources allows both linear and non-linear coregistration of multimodal acquisitions performed on the same animal (functional, anatomical and diffusion), leading to an accurate identification of the brain territories involved in a physiological or pathophysiological process. Therefore, the SIGMA resources pave the way for standardization in preclinical research, but also for multicentric preclinical studies needed to increase their significance.

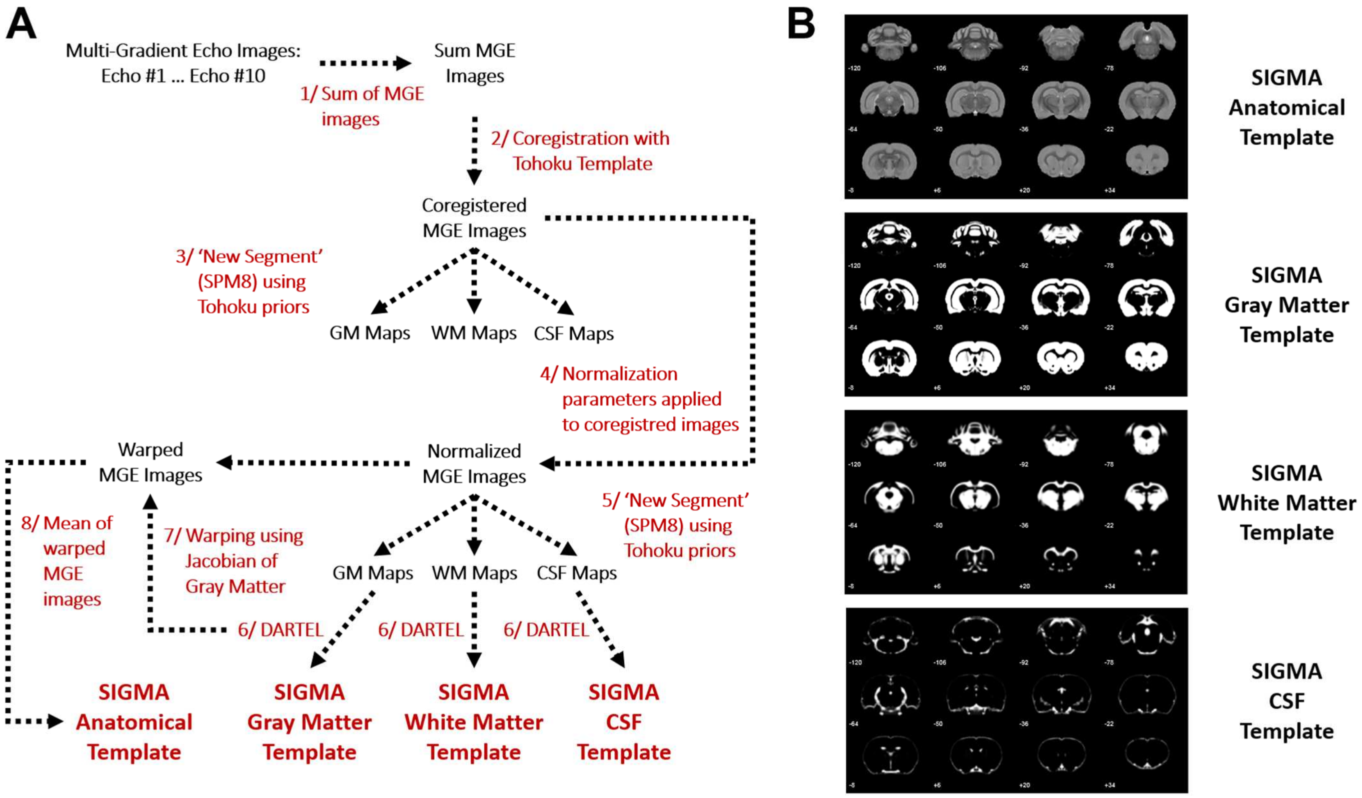

Supplementary Figure 5: (**A**) Schematic workflow used for the creation of (**B**) the *ex vivo* SIGMA anatomical template of rat brain and the corresponding Gray Matter (GM), White Matter (WM) and Cerebrospinal Fluid (CSF) probability maps.

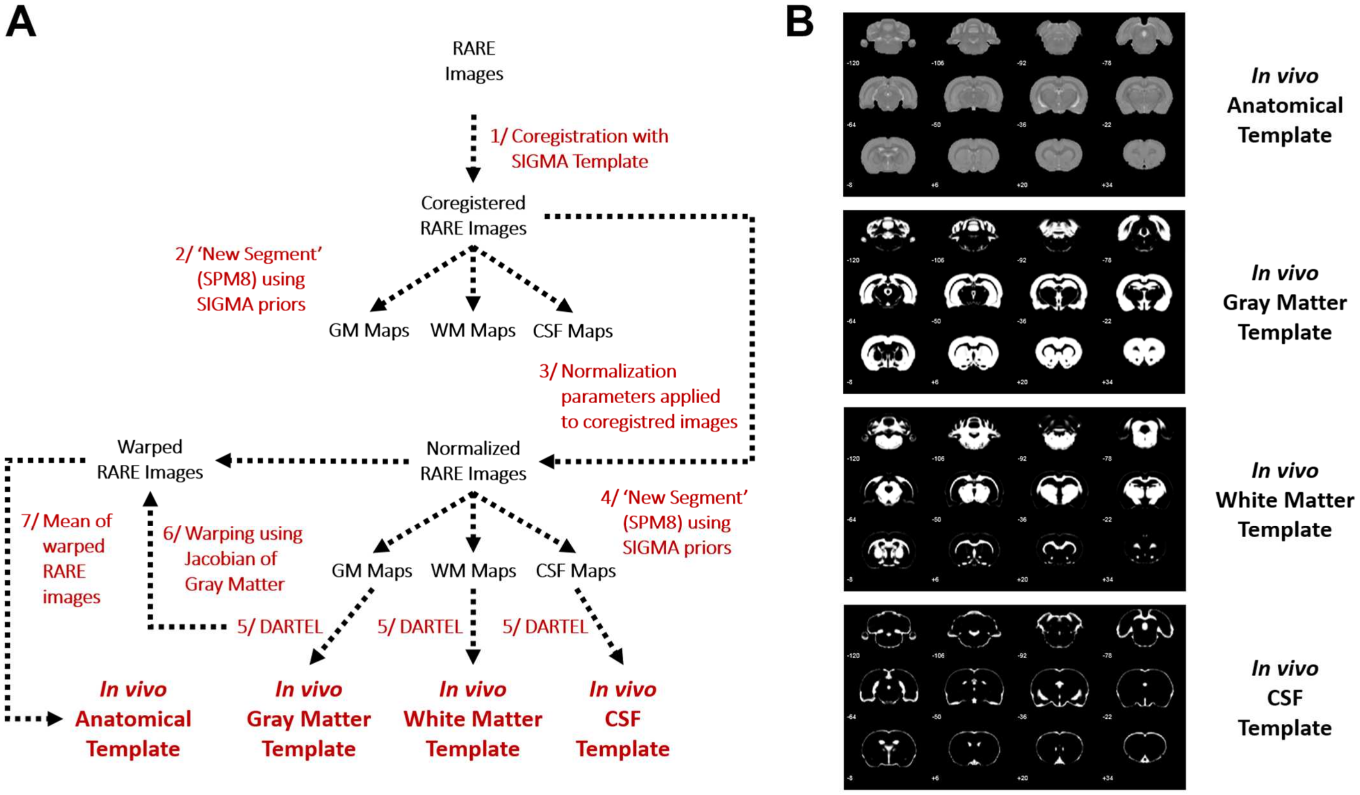

Supplementary Figure 6: (**A**) Schematic workflow used for the creation of (**B**) the *in vivo* anatomical template of rat brain and the corresponding Gray Matter (GM), White Matter (WM) and Cerebrospinal Fluid (CSF) probability maps.

Supplementary Table 1: Listing of anatomical regions of interest from the SIGMA anatomical atlas.

Original Atlas	Left Hemisphere Label	Right Hemisphere Label	Matters	Territories	Systems	Regions of interest
SIGMA	1171	1172	CSF	CSF	CSF	Ventricular System
Waxholm	171	172	Grey Matter	Basal ganglia	Pallidum	Globus Pallidus
Waxholm	731	732	Grey Matter	Basal ganglia	Striatal System	Striatum
Waxholm	221	222	Grey Matter	Cerebellum	Sensory-Motor	Granule Cell Level of the Cerebellum
Waxholm	341	342	Grey Matter	Cerebellum	Sensory-Motor	Molecular Layer of the Cerebellum
Tohoku	31	32	Grey Matter	Cortex	Amygdala	Amygdalohyppocampic Area
Tohoku	41	42	Grey Matter	Cortex	Amygdala	Amygdalopiriform Cortex
Tohoku	491	492	Grey Matter	Cortex	Auditory System	Primary Auditory Cortex
Tohoku	681	682	Grey Matter	Cortex	Auditory System	Secondary Auditory Cortex Dorsal Part
Tohoku	691	692	Grey Matter	Cortex	Auditory System	Secondary Auditory Cortex Ventral Part
SIGMA	211	212	Grey Matter	Cortex	Auditory System	Lateral Primary Auditory Cortex
Tohoku	501	502	Grey Matter	Cortex	Cingular System	Primary Cingular Cortex
Tohoku	701	702	Grey Matter	Cortex	Cingular System	Secondary Cingular Cortex
Waxholm	71	72	Grey Matter	Cortex	Hippocampus Fomation	Cornu Ammonis 1
Waxholm	81	82	Grey Matter	Cortex	Hippocampus Fomation	Cornu Ammonis 2
Waxholm	91	92	Grey Matter	Cortex	Hippocampus Fomation	Cornu Ammonis 3
Waxholm	101	102	Grey Matter	Cortex	Hippocampus Fomation	Dentate Gyrus
Waxholm	151	152	Grey Matter	Cortex	Hippocampus Fomation	Fasciola Cinereum
Waxholm	371	372	Grey Matter	Cortex	Hippocampus Fomation	Parasubiculum
Waxholm	471	472	Grey Matter	Cortex	Hippocampus Fomation	Presubiculum
Waxholm	741	742	Grey Matter	Cortex	Hippocampus Fomation	Subiculum
Tohoku	11	12	Grey Matter	Cortex	Insular System	Agranular Dysgranular Insular Cortex
Tohoku	21	22	Grey Matter	Cortex	Insular System	Agranular Insular Cortex
Tohoku	121	122	Grey Matter	Cortex	Insular System	Dysgranular Insular Cortex
Tohoku	451	452	Grey Matter	Cortex	Insular System	Posterior Agralunar Insular Cortex
Tohoku	111	112	Grey Matter	Cortex	Limbic System	Dorso Lateral Orbital Cortex
Tohoku	161	162	Grey Matter	Cortex	Limbic System	Frontal Association Cortex
SIGMA	361	362	Grey Matter	Cortex	Limbic System	Orbitofrontal Region
SIGMA	461	462	Grey Matter	Cortex	Limbic System	PreLimbic System
Tohoku	511	512	Grey Matter	Cortex	Motor System	Primary Motor Cortex
Tohoku	711	712	Grey Matter	Cortex	Motor System	Secondary Motor Cortex
Waxholm	141	142	Grey Matter	Cortex	Olfactory System	Entorhinal Cortex
Tohoku	251	252	Grey Matter	Cortex	Olfactory System	Lateral Entorhinal Cortex Internal part
Tohoku	261	262	Grey Matter	Cortex	Olfactory System	Lateral Entorhinal Cortex
Tohoku	271	272	Grey Matter	Cortex	Olfactory System	Lateral Entorhinal Cortex external part
Tohoku	301	302	Grey Matter	Cortex	Olfactory System	Medial Entorhinal Cortex
Waxholm	421	422	Grey Matter	Cortex	Olfactory System	Perirhinal Area 35
Waxholm	431	432	Grey Matter	Cortex	Olfactory System	Perirhinal Area 36
Tohoku	441	442	Grey Matter	Cortex	Olfactory System	Perirhinal Cortex
Tohoku	281	282	Grey Matter	Cortex	Parietal System	Lateral Parietal Associative Cortex
Tohoku	311	312	Grey Matter	Cortex	Parietal System	Medial Parietal Associative Cortex
Tohoku	381	382	Grey Matter	Cortex	Parietal System	Parietal Cortex Postero Caudal Part
Tohoku	391	392	Grey Matter	Cortex	Parietal System	Parietal Cortex Postero Dorsal Part
Tohoku	401	402	Grey Matter	Cortex	Parietal System	Parietal Cortex Postero Rostral
Tohoku	651	652	Grey Matter	Cortex	Retrosplenial System	Retosplenial Dysgranular Cortex
Tohoku	661	662	Grey Matter	Cortex	Retrosplenial System	Retrosplenial Granular Cortex Part A
Tohoku	671	672	Grey Matter	Cortex	Retrosplenial System	Retrosplenial Granular Cortex Part B
Tohoku	521	522	Grey Matter	Cortex	Somatosensory System	Primary Somatosensory Cortex Barrel field
Tohoku	531	532	Grey Matter	Cortex	Somatosensory System	Primary Somatosensory Cortex Dysgranular
Tohoku	541	542	Grey Matter	Cortex	Somatosensory System	Primary Somatosensory Cortex Dysgranular Zone 0
Tohoku	551	552	Grey Matter	Cortex	Somatosensory System	Primary Somatosensory Cortex Forelimb
Tohoku	561	562	Grey Matter	Cortex	Somatosensory System	Primary Somatosensory Cortex Hindlimb
Tohoku	571	572	Grey Matter	Cortex	Somatosensory System	Primary Somatosensory Cortex Jaw
Tohoku	581	582	Grey Matter	Cortex	Somatosensory System	Primary Somatosensory Cortex
Tohoku	591	592	Grey Matter	Cortex	Somatosensory System	Primary Somatosensory Cortex Shoulder
Tohoku	601	602	Grey Matter	Cortex	Somatosensory System	Primary Somatosensory Cortex Trunk
Tohoku	611	612	Grey Matter	Cortex	Somatosensory System	Primary Somatosensory Cortex Upperlips
Tohoku	721	722	Grey Matter	Cortex	Somatosensory System	Secondary Somatosensory Cortex
Tohoku	131	132	Grey Matter	Cortex	Temporal System	Ectorhinal Cortex
SIGMA	201	202	Grey Matter	Cortex	Temporal System	Lateral Temporal Associative Cortex
Tohoku	771	772	Grey Matter	Cortex	Temporal System	Temporal Associative Cortex
Tohoku	291	292	Grey Matter	Cortex	Visual System	Lateral Secondary Visual Cortex
Tohoku	321	322	Grey Matter	Cortex	Visual System	Medio Lateral Secondary Visual Cortex
Tohoku	331	332	Grey Matter	Cortex	Visual System	Medio Medial Secondary Visual Cortex
Tohoku	621	622	Grey Matter	Cortex	Visual System	Primary Visual Cortex Binocular Area
Tohoku	631	632	Grey Matter	Cortex	Visual System	Primary Visual Cortex
Tohoku	641	642	Grey Matter	Cortex	Visual System	Primary Visual Cortex Monocular Area
Waxholm	61	62	Grey Matter	Diencephalon	Thalamus	Bed Nucleus of the Stria Terminalis
Waxholm	231	232	Grey Matter	Diencephalon	Hypothalamus	Hypothalamic Region
Waxholm	411	412	Grey Matter	Diencephalon	Tegmentum	Periaqueductal Gray
Waxholm	481	482	Grey Matter	Diencephalon	Visual System	Pretectal Region
Waxholm	971	972	Grey Matter	Diencephalon	Medulla	External Cortex of the Inferior Colliculus
Waxholm	1061	1062	Grey Matter	Diencephalon	Septum	Septal Region
Waxholm	51	52	Grey Matter	Forebrain	Forebrain	Basal Forebrain Region
SIGMA	241	242	Grey Matter	Mesencephalon	Tegmentum	Interpeduncular Nucleus
Waxholm	751	752	Grey Matter	Mesencephalon	Tegmentum	Substantia Nigra
Waxholm	761	762	Grey Matter	Mesencephalon	Colliculi	Superficial Gray Layer of the Superior Colliculus
Waxholm	181	182	Grey Matter	Olfactive Bulb	Olfactory System	Glomerular Layer of the Accessory Olfactory Bulb
Waxholm	191	192	Grey Matter	Olfactive Bulb	Olfactory System	Glomerular Layer of the Olfactory Bulb
Waxholm	351	352	Grey Matter	Olfactive Bulb	Olfactory System	Olfactory Bulb
Waxholm	801	802	White Matter	Basal ganglia	Inter Hemispheric Commisures	Anterior Commissure anterior part
Waxholm	811	812	White Matter	Basal ganglia	Inter Hemispheric Commisures	Anterior Commissure posterior part
Waxholm	1091	1092	White Matter	Basal ganglia	Inter Hemispheric Commisures	Dorsal Fornix
Waxholm	821	822	White Matter	Brainstem	Sensory System	Ascending Fibers of the Facial Nerve
Waxholm	841	842	White Matter	Brainstem	Medulla	Brainstem
Waxholm	851	852	White Matter	Brainstem	Medulla	Central Canal
Waxholm	921	922	White Matter	Brainstem	Sensory System	Genu of the Facial Nerve
Waxholm	1031	1032	White Matter	Brainstem	Medulla	Inferior Olive/PyramidalDecusation/SP5
Waxholm	1041	1042	White Matter	Brainstem	Medulla	Posterior Commissure
Waxholm	1101	1102	White Matter	Brainstem	Medulla	Fimbria of the Hippocampus part 1
Waxholm	1161	1162	White Matter	Brainstem	Medulla	Periventricular Grey
Waxholm	991	992	White Matter	Cerebellum	Spinocerebellar Pathway	Medial Lemniscus Decussation
Waxholm	1001	1002	White Matter	Cerebellum	Spinocerebellar Pathway	Medial Lemniscus
Waxholm	1011	1012	White Matter	Cerebellum	Somatosensory System	Middle Cerebellar Peduncle
Waxholm	1051	1052	White Matter	Cerebellum	Cerebellopontine Pathway	Pyramidal tract
Waxholm	781	782	White Matter	Cortex	Hippocampus Fomation	Fimbria of the Hippocampus part 2
Waxholm	941	942	White Matter	Cortex	Hippocampus Fomation	Fimbria of the Hippocampus part 3
Waxholm	961	962	White Matter	Cortex	Inter Hemispheric Commisures	Inferior Cerebellar Peduncle/Spinal Trigeminal Tract
Waxholm	861	862	White Matter	Diencephalon	Thalamus	Commissural Stria Terminalis

Waxholm	951	952	White Matter	Diencephalon	Thalamus	Fornix
Waxholm	981	982	White Matter	Diencephalon	Inter Hemispheric Commisures	Mammillothalamic Tract
Waxholm	1021	1022	White Matter	Diencephalon	Thalamus	Optic Tract and Optic Chiasm
Waxholm	1071	1072	White Matter	Diencephalon	Sensory System	Spinal Cord
Waxholm	1111	1112	White Matter	Diencephalon	Thalamus	Subthalamic Nucleus
Waxholm	1131	1132	White Matter	Diencephalon	Thalamus	Thalamus
Waxholm	1141	1142	White Matter	Diencephalon	Pons	Brachium Pontis
Waxholm	1151	1152	White Matter	Diencephalon	Thalamus	Ventral Hippocampal Commissure
Waxholm	891	892	White Matter	Fiber tract	Corpus Callosum	Corpus Callosum and Associated Subcortical White Matter
Waxholm	911	912	White Matter	Fiber tract	Internal Capsule	Descending Corticofugal Pathways and Globus Pallidum
Waxholm	931	932	White Matter	Fiber tract	Thalamus	Fasciculus Retroflexus
Waxholm	831	832	White Matter	Mesencephalon	Colliculi	Brachium of the Superior Colliculus
Waxholm	871	872	White Matter	Mesencephalon	Colliculi	Commissure of the Inferior Colliculus
Waxholm	881	882	White Matter	Mesencephalon	Colliculi	Commissure of the Superior Colliculus
Waxholm	901	902	White Matter	Mesencephalon	Inter Hemispheric Commisures	Deeper Layers of the Superior Colliculus
Waxholm	1081	1082	White Matter	Mesencephalon	Medulla	SP5
Waxholm	791	792	White Matter	Olfactory Bulb	Inter Hemispheric Commisures	Anterior Commissure Intrabulbar part
Waxholm	1121	1122	White Matter	Spinal Cord	Fiber tract	Optic Pathways

Supplementary Table 2: Effect of the normalization procedure onto the volumes of both Tohoku and Waxholm regions of interest observed when building the SIGMA anatomical atlas.

Atlas	ROI Names	ROI volumes before normalization (µL)	ROI volumes after normalization (µL)	Variation (%)
WHS	anterior commissure, anterior part	2.00	2.01	0.50
WHS	anterior commissure, intrabulbar part	2.00	2.71	35.30
WHS	anterior commissure, posterior part	1.00	0.39	-61.20
WHS	ascending fibers of the facial nerve	1.00	0.19	-81.40
WHS	basal forebrain region	80.00	81.63	2.03
WHS	bed nucleus of the stria terminalis	2.00	2.62	30.90
WHS	brachium of the superior colliculus	1.00	0.39	-61.20
WHS	brain stem	209.00	228.80	9.47
WHS	central canal	1.00	0.22	-77.60
WHS	commissural stria terminalis	1.00	1.00	0.00
WHS	commissure of the inferior colliculus	6.00	6.93	15.55
WHS	commissure of the superior colliculus	0.16	0.18	15.00
WHS	cornu ammonis 1	27.00	33.54	24.21
WHS	cornu ammonis 2	5.00	5.38	7.60
WHS	cornu ammonis 3	32.00	29.90	-6.55
WHS	corpus callosum and associated subcortical white matter	79.00	80.74	2.20
WHS	corticofugal pathways	30.00	43.67	45.57
WHS	deeper layers of the superior colliculus	24.00	30.59	27.48
WHS	dentate gyrus	32.00	46.79	46.23
WHS	entorhinal cortex	24.00	19.86	-17.25
WHS	fasciculus retroflexus	1.00	0.19	-81.20
WHS	fasciola cinereum	3.00	3.71	23.57
WHS	fimbria of the hippocampus	11.00	4.82	-56.21
WHS	fornix	3.00	3.59	19.53
WHS	genu of the facial nerve	1.00	0.49	-51.40
WHS	globus pallidus	0.20	0.18	-9.00
WHS	glomerular layer of the accessory olfactory bulb	2.00	0.82	-59.00
WHS	glomerular layer of the olfactory bulb	20.00	28.28	41.40
WHS	hypothalamic region	24.00	31.42	30.90
WHS	inferior cerebellar peduncle	60.00	18.70	-68.84
WHS	inferior olive	20.00	13.25	-33.76
WHS	interpeduncular nucleus	10.00	3.82	-61.80
WHS	mammillothalamic tract	1.00	1.28	28.40
WHS	medial lemniscus	2.00	1.35	-32.30
WHS	medial lemniscus decussation	1.00	0.13	-86.60
WHS	middle cerebellar peduncle	10.00	10.44	4.36
WHS	molecular cell layer of the cerebellum	166.00	262.77	58.30
WHS	olfactory bulb	122.00	112.15	-8.08
WHS	optic tract and optic chiasm	6.00	9.21	53.47
WHS	parasubiculum	8.00	10.90	36.20
WHS	periaqueductal gray	17.00	25.36	49.18
WHS	perirhinal area 35	6.00	6.91	15.10
WHS	perirhinal area 36	9.00	14.79	64.31
WHS	periventricular gray	13.00	11.85	-8.82
WHS	posterior commissure	1.00	0.32	-68.00
WHS	presubiculum	9.00	1.34	-85.09
WHS	pretectal region	8.00	2.32	-71.03
WHS	pyramidal decussation	1.00	0.26	-74.00
WHS	septal region	12.00	11.25	-6.23
WHS	striatum	111.00	102.85	-7.34
WHS	subiculum	17.00	17.29	1.69
WHS	substantia nigra	7.00	4.05	-42.20
WHS	subthalamic nucleus	1.00	0.28	-71.80
WHS	superficial gray layer of the superior colliculus	7.00	10.25	46.49
WHS	thalamus	86.00	60.80	-29.31
WHS	ventral hippocampal commissure	1.00	0.92	-7.80
Tohoku	AIP	7.73	8.66	12.11
Tohoku	APir	13.04	1.74	-86.68
Tohoku	Au1	20.08	28.78	43.30
Tohoku	AUD	8.93	12.91	44.65
Tohoku	AuV	6.38	8.49	33.06
Tohoku	Cg1	24.91	34.80	39.73
Tohoku	Cg2	7.65	9.83	28.41
Tohoku	DI	8.99	14.25	58.57
Tohoku	DIEnt	6.98	7.86	12.58
Tohoku	DLEnt	20.11	2.37	-88.22
Tohoku	DLO	4.87	6.19	27.03
Tohoku	Ect	18.64	21.69	16.38
Tohoku	Fr3	9.60	5.68	-40.85

Tohoku	LPtA	8.23	11.39	38.38
Tohoku	M1	43.33	47.48	9.56
Tohoku	M2	24.08	22.76	-5.46
Tohoku	MEnt	6.29	4.73	-24.71
Tohoku	MPtA	3.25	4.18	28.62
Tohoku	PRh	1.43	2.18	51.88
Tohoku	PtPC	19.00	6.64	-65.05
Tohoku	PtPD	4.78	0.30	-93.76
Tohoku	PtPR	1.35	2.01	49.26
Tohoku	RSD	22.77	38.12	67.42
Tohoku	RSGa	3.01	5.15	70.96
Tohoku	RSGb	4.98	7.46	49.73
Tohoku	S1	1.98	2.77	39.82
Tohoku	S1BF	36.77	44.65	21.42
Tohoku	S1DZ	13.29	16.63	25.11
Tohoku	S1DZ0	4.30	6.63	54.29
Tohoku	S1FL	19.62	23.87	21.66
Tohoku	S1HL	9.43	11.40	20.92
Tohoku	S1J	8.10	11.58	42.99
Tohoku	S1Sh	0.53	0.67	26.42
Tohoku	S1Tr	2.99	3.96	32.60
Tohoku	S1ULp	22.69	32.67	43.98
Tohoku	S2	9.59	13.79	43.71
Tohoku	TeA	6.45	8.49	31.55
Tohoku	V1	9.05	12.54	38.67
Tohoku	V1B	17.14	25.26	47.36
Tohoku	V1M	10.02	15.76	57.30
Tohoku	V2L	8.63	12.35	43.18
Tohoku	V2ML	6.00	8.28	38.06
Tohoku	V2MM	2.22	3.11	39.66

**Global mean of volume variations (%)**	**+ 3.59**
**Mean of absolute volume variations (%)**	**38.54**
**Standard deviation of absolute volume variations (%)**	**24.04**

Supplementary Table 3: Listing of functional regions of interest from the SIGMA functional atlas.

Label	Hemisphere	Territories	System	Region of interest
7	Inter hemispheric	Basal ganglia	Striatal system	Accumbens Shell
13	Left	Basal ganglia	Striatal system	Striatum (Dorsal)
14	Left	Basal ganglia	Striatal system	Striatum (Ventral)
15	Right	Basal ganglia	Striatal system	Striatum
8	Right	Cortex	Motor Cortex	Primary and Secondary Motor
10	Right	Cortex	Amygdala	Amygdala (Central, Basolateral)
1	Inter hemispheric	Cortex	Cingular system	Cingulate Cortex 1
9	Inter hemispheric	Cortex	Cingular system	Cingulate Cortex 2
18	Inter hemispheric	Cortex	Cingular system	Cingulate Cortex 3
23	Left	Cortex	Hippocampus Fomation	Dorsal Hippocampus
32	Left	Cortex	Hippocampus Fomation	Cornu Ammonis 1 (transition dorsal ventral)
36	Right	Cortex	Hippocampus Fomation	Dorsal Hippocampus
40	Left	Cortex	Hippocampus Fomation	Dorsal Dentate Gyrus
48	Left	Cortex	Hippocampus Fomation	Cornu Ammonis 1 (Ventral)
44	Right	Cortex	Hippocampus Fomation	Dorsal Dentate Gyrus/Entorhinal Cortex
6	Right	Cortex	Insular system	Insular Cortex
17	Right	Cortex	Insular system	Insular Cortex 2
20	Left	Cortex	Insular system	Insular Cortex
2	Inter hemispheric	Cortex	Limbic System	Prelimbic Cortex
5	Inter hemispheric	Cortex	Limbic System	Prelimbic/Infralimbic
16	Left	Cortex	Olfactory system	Endo/Piriform Cortex
22	Right	Cortex	Olfactory system	Piriform Cortex
26	Left	Cortex	Olfactory system	Piriform Cortex
37	Right	Cortex	Olfactory system	Intermedial Entorhinal cortex
25	Inter hemispheric	Cortex	Retrosplenial system	Retrosplenial Cortex 1
28	Inter hemispheric	Cortex	Retrosplenial system	Retrosplenial Granular Cortex c
38	Inter hemispheric	Cortex	Retrosplenial system	Retrosplenial Cortex 2
41	Inter hemispheric	Cortex	Retrosplenial system	Retrosplenial Cortex 3/Superior Gray
45	Inter hemispheric	Cortex	Retrosplenial system	Retrosplenial Cortex 4
49	Left	Cortex	Retrosplenial system	Retrosplenial Granular Cortex Zone A/Postsubiculum
50	Right	Cortex	Retrosplenial system	Retrosplenial Granular Cortex Zone A/Postsubiculum
51	Inter hemispheric	Cortex	Retrosplenial system	Retrosplenial Cortex 5/Colliculus
58	Right	Cortex	Retrosplenial system	RSD/RSGa
3	Right	Cortex	Somatosensory system	Primary Somatosensory
4	Left	Cortex	Somatosensory system	Primary Somatosensory
11	Right	Cortex	Somatosensory system	Primary Somatosensory Cortex (BE)
12	Left	Cortex	Somatosensory system	Primary Somatosensory Cortex (BE)
24	Right	Cortex	Somatosensory system	Primary Somatosensory Cortex (Auditory)
29	Left	Cortex	Somatosensory system	Parietal Cortex (Auditory)
34	Right	Cortex	Somatosensory system	Parietal Cortex (Auditory)
31	Inter hemispheric	Cortex	Visual system	Secondary Visual cortex
39	Left	Cortex	Visual system	Primary and Secondary Visual Cortex
42	Right	Cortex	Visual system	Primary and Secondary Visual Cortex
21	Inter hemispheric	Diencephalon	Hypothalamus	Hypothalamus 1
30	Inter hemispheric	Diencephalon	Hypothalamus	Hypothalamus 2
33	Inter hemispheric	Diencephalon	Tegmentum	Dorsal Lateral Periaqueductal Gray Left
19	Right	Diencephalon	Thalamus	Dorsal thalamic nucleus
27	Inter hemispheric	Diencephalon	Thalamus	Ventral thalamic
35	Left	Hind Brain	Visual system	Medial geniculate nucleus
43	Inter hemispheric	Mesencephalon	Tegmentum	Interpeduncular nucleus
46	Right	Mesencephalon	Colliculi	Inferior colliculus/external cortex
47	Inter hemispheric	Mesencephalon	Pons	Ventral tegmental nucleus
52	Inter hemispheric	Mesencephalon	Pons	Pontine nuclei
53	Inter hemispheric	Mesencephalon	Pons	Raphe (pallidum/magnun) nuclei
54	Left	Mesencephalon	Pons	Pontine reticular/subcoeruleus
55	Left	Mesencephalon	Colliculi	External colliculus (V1)
56	Right	Mesencephalon	Pons	Subcoeruleum/pontine reticular nucleu
57	Right	Mesencephalon	Colliculi	Colliculus
59	Right	Mesencephalon	Pons	Raphe/Median(paramedian) pontine reticular nu
